# Draft Genome Sequence of a Methicillin-Resistant Sequence Type 39 Staphylococcal Isolate Obtained from Seafood

**DOI:** 10.1128/genomeA.01193-17

**Published:** 2017-11-09

**Authors:** G. K. Sivaraman, Deesha Vanik, S. Visnuvinayagam, Mothadaka Mukteswar Prasad, V. Murugadas, Ranjit Kumar Nadella, C. N. Ravishankar

**Affiliations:** a Regional Centre of ICAR-Central Institute of Fisheries Technology, Veraval, Gujarat State, India; b Research Centre of ICAR-Central Institute of Fisheries Technology, Vashi, Navi Mumbai, Maharashtra, India; c ICAR-Central Institute of Fisheries Technology, Willingdon Island, Cochin, Kerala State, India

## Abstract

The draft genome sequence of a methicillin-resistant *Staphylococcus aureus* (MRSA) sequence type 39 (ST 39) isolate obtained from the dried ribbonfish of Gujarat, India, is reported here. *Staphylococcus*-specific genes were present in this MRSA isolate. The whole-genome sequence of this strain contains 2,693 protein-coding genes and 70 RNAs within the 2.82-Mb genome.

## GENOME ANNOUNCEMENT

*Staphylococcus aureus* is a ubiquitous organism present on the skin surfaces and anterior nares of 25% of healthy people and animals as carriers, but it does not cause illness to healthy individuals. It is a well-known opportunistic pathogen causing mild skin infections to invasive and toxin-mediated diseases (1–3). Staphylococcal species are not members of the normal microflora of fish. A subset of *S. aureus* strains have developed resistance to oxacillin antibiotics by way of acquiring a mobile staphylococcal chromosomal cassette (SCC) carrying the methicillin resistance gene *mecA* (4). Methicillin-resistant *Staphylococcus aureus* (MRSA) is internationally acknowledged as a zoonotic multidrug-resistant pathogen (5). Numerous studies have reported the occurrence of MRSA in farms and food-producing animals in Europe, the United States, and Asia (6). The incidence of MRSA in fish and other seafood has recently been noted (7–9). MRSA is possibly introduced by healthy carriers, fish handlers, contaminated surfaces, water, and ice during processing, transport, and storage (10).

The genome of the MRSA isolate with multilocus sequence type (MLST)/*spa* type ST39/t007 (MRSARF-10) was commercially sequenced to determine the genetic structure of its multiple-drug resistance genes. Preenrichment was done on tryptic soy broth (Oxoid, United Kingdom), followed by incubation at 35°C for 18 to 24 h, and a loopful of culture was streaked onto a MRSA II agar plate (Difco, USA). DNA was extracted from a typical mauve-colored colony using a genomic DNA isolation kit (Sigma, France). The Illumina HiSeq 2500 platform was used for whole-genome sequencing of this clone. The number of paired-end reads was approximately 7 billion short-read sequences in pairs of ~300 bp, the number of bases was 650.26 Mb, and the G+C content was 35.28%. *De novo* contig assembly was performed using MaSuRCA (11), and further downstream processing was performed. Coding sequences (CDSs) were predicted from the contigs using Glimmer (12), and 2,693 predicted CDSs were found. The predicted CDSs were annotated using the in-house pipeline CANoPI (Contig Annotator Pipeline) in comparison with the NCBI database using the BLASTX program. Organism annotation, gene and protein annotation to the matched genes, gene ontology annotation, and pathway annotation were carried out with the use of the NCBI database. Matches with an *E* value of ≤10^−5^ and similarity score of ≥50% were retained for further annotation. Overall, we observed that 2,669 (99.10%) of the predicted CDSs had at least one hit in the NCBI database. Nearly 87% of the CDSs found using BLASTX have a confidence level of at least 1 × 10^−5^, which indicates high protein conservation. We found that 100% of the predicted CDSs have a similarity of more than 60% at the protein level with the existing proteins at the NCBI database. The majority of the top BLASTX hits belong to MRSA (top 15 organisms). Among the total significant BLASTX hit CDSs, 1,703 genes were annotated using the UniProt database. The total number of Gene Ontology annotations identified for molecular functions was 889, with 604 annotations having to do with a biological process and 236 annotations having to do with cellular components. We predicted tRNA genes from the contigs using tRNAscan-SE (13) and found 70 genes.

### Accession number(s).

This whole-genome shotgun project has been deposited at NCBI GenBank under the accession number NBZY00000000.
